# Differences in heart rate variability may be related to the appearance of postoperative pain in patients undergoing breast cancer surgery

**DOI:** 10.1186/s40981-017-0123-4

**Published:** 2017-10-11

**Authors:** Shinya Uchida, Yuji Kadoi, Shigeru Saito

**Affiliations:** 1 Department of Anesthesia, Gunma Cancer Center, Maebashi, Japan; 2 Department of Anesthesiology, Gunma University Hospital, 3-39-22 Showa-Machi, Maebashi, Gunma 371-8511 Japan

**Keywords:** Heart rate variability, Predictable tool, Postoperative pain

## Abstract

**Background:**

Some reports have highlighted the relationship between heart rate variability (HRV) and the degree of postoperative pain experienced. This study retrospectively examined whether differences in heart rate variability may be related to the appearance of postoperative pain in patients undergoing breast cancer surgery.

**Findings:**

We retrospectively analyzed 20 postoperative patients who had no pain immediately upon admission to the post-anesthesia care unit (PACU), divided into two groups: group A (*n* = 16) had no pain on admission to PACU, remaining pain free upon discharge (12 h after surgery); group B (*n* = 4) comprised patients with no pain on admission to PACU but who experienced increasing pain requiring intervention in PACU 1 h after surgery. HRV was measured immediately on admission to PACU and 2 h after surgery; this included variables of low-frequency power (LF), high-frequency power (HF), and LF/HF. There were significant differences in HF and LF/HF in group A compared with those in group B on admission to PACU (immediately after arrival): HF, group A, 35.4 ± 18.1; group B, 64.2 ± 9.5*; LF/HF group A, 2.7 ± 2.4; group B, 0.6 ± 0.2*, *p < 0.05). There was no significant difference in the Numerical rating scale (NRS) between the two groups immediately after admission to PACU. At 1 h after the surgery, NRS in Group B increased, and there were significant differences in NRS values between the two groups 1 h after surgery prior to the use of analgesic agents (NRS, group A, 1.0 ± 0.9; group B, 4.0 ± 1.4*, *p < 0.01). Patients in group A required no analgesic agents for at least 12 h after surgery.

**Conclusions:**

Lower HF and higher LF/HF values immediately after arrival in PACU were observed in patients remaining pain free for 12 h after surgery compared to patients who experienced increasing postoperative pain 1 h after surgery. The data suggest that differences in HRV may be related to the appearance of postoperative pain.

## Introduction

The measurement of heart rate variability (HRV) is a non-invasive method for assessing sympathetic and parasympathetic nervous system activity [1]. Spectral analysis of the ECG makes it possible to show the relationship between parasympathetic and sympathetic tone in different clinical situations. It has been shown that the peak of the high-frequency (HF) component reflects parasympathetic activity, whereas the peak of the low-frequency (LF) component depends on the balance between sympathetic and parasympathetic tone.

Some reports have investigated the relationship between HRV and pain intensity as a predictive tool [2–8]. Jeanne et al. [2] examined the impact of both surgical nociception and analgesia on HRV in anesthetized patients and showed that a decrease in high-frequency power was an indicator of insufficient depth of anesthesia. Subsequently, Papaioannou et al. [3] demonstrated that the balance between analgesia and nociception calculated from HRV predicted pain perception in burns patients. Lindh et al. [8] reported that HRV was a possible predictor of pain in newborn infants. These reports suggest that perioperative assessment of HRV could be a useful tool for predicting postoperative pain.

We hypothesized that assessing HRV in the post-anesthesia care unit (PACU) could predict the appearance of postoperative pain in surgical patients. Until now, there have been no reports examining the relationship between changes in HRV and postoperative pain. The purpose of this study was to retrospectively examine whether differences in heart rate variability may be related to the appearance of postoperative pain in patients undergoing breast cancer surgery.

## Methods

The study protocols were approved by the ethics committee of the hospital (IRB405-27019). Written, informed consent was obtained from all patients prior to participation. We analyzed data from all 82 patients who underwent breast cancer surgery between 2015 and 2016 (University Hospital Medical Information Network (UMIN) Clinical Trials Registry, UMIN registration 000023285).

We selected patients who underwent breast cancer surgery because the procedures involved similar surgical time and were performed by the same surgeons, so that acute postoperative surgical pain experienced was thought to be similar in these patients.

Of the 82 patients, 20 were excluded from the study because their postoperative pain scores on admission to PACU necessitated treatment with continuous low-dose remifentanil; their NRS values were above 4. Sixteen additional patients were excluded because of inadequate HRV data, due to noise from postoperative shivering interfering with HRV measurement. Patients were also excluded if they were elderly (over 70 years), had a history of cerebrovascular disease and ischemic heart disease, were treated with beta-blockers, had chronic obstructive lung disease, psychiatric illness, and/or active liver disease (the latter defined as serum glutamine oxaloacetate transaminase (GOT) or glutamine pyruvate transaminase (GPT) levels above 50 U/dL). After exclusions, 20 out of 82 patients could be analyzed. Of these 82 patients, we were able to retrospectively select 20 patients who had no pain after surgery on admission to the post-anesthesia care unit (PACU); “no pain” defined as a numerical rating scale (NRS) value between 0 and 2.

These 20 patients were further divided into two groups. Group A (*n* = 16) had no pain upon admission to PACU and remained pain free upon discharge from PACU (12 h after surgery); group B (*n* = 4) had no pain upon admission to PACU, but experienced increasing pain in PACU 1 h after surgery, requiring analgesic therapy (Table 1).

**Table 1:** Study protocol

1. The study sample comprised 82 patients who underwent breast cancer surgery between 2015 and 2016 in our institution.
2. Patients were excluded if they met our exclusion criteria.
3. We then selected 20 patients who had no pain after surgery immediately upon admission to the post-anesthesia care unit (PACU) (with “no pain” defined as a Numerical Rating Scale (NRS) value between 0 and 2).
4. These 20 patients were divided into two groups: Group A (*n* = 16) had no pain upon admission to PACU and remained pain free upon discharge from PACU (12 h after surgery); group B (*n* = 4) had no pain immediately upon admission to PACU and experienced increasing pain 1 h after surgery while in PACU, requiring analgesic therapy.
5. NRS was measured immediately upon arrival in PACU, then 1 and 2 h after surgery.
6. Heart rate variability (HRV) was measured upon arrival in PACU and then 2 h after surgery.
7. These 20 patients (both group A (*n* = 16) and group B (*n* = 4) remained in PACU for 12 h after surgery.

Anesthetic technique comprised intravenous induction using 2 mg/kg of propofol, 1.0 mg/kg of rocuronium, and 0.2–0.5 μg/kg/min of remifentanil, followed by endotracheal intubation. Muscle relaxation was achieved using intermittent administration of rocuronium. All patients were mechanically ventilated using a mixture of 50% oxygen and 50% N_2_ with continuous monitoring of end-tidal carbon dioxide. Anesthesia was maintained with a remifentanil infusion of 0.2–0.4 μg/kg/min and inhalation of 1.0–2.0% sevoflurane in 50% oxygen and 50% N_2_. During surgery, the sevoflurane concentration was adjusted by the anesthesiologist to ensure appropriate hemodynamics.

Following conclusion of surgery but before emergence from anesthesia, the “pecs” block was performed under ultrasound guidance in the operating room (infiltration 20 mL of 0.375% ropivacaine into the interfascial plane between the pectoralis major and minor muscles) [9]. All anesthetic and analgesic agents were discontinued after surgery concluded, and patients were extubated awake. Immediately following extubation, all patients were then transferred to PACU where HRV monitoring was instituted. Transfer time from the operating room to PACU was about 5 min.

Anesthesiologists analyzed patients’ pain using the numeric rating scale (NRS; 0–10). If the NRS was above 4, additional analgesic agents were used if patients complained of pain. Patients who required additional analgesic agents immediately after surgery upon arrival at PACU were excluded from the data analysis. In these patients, non-steroidal anti-inflammatory drugs (NSAIDs; flurbiprofen axetil 50 mg) or continuous infusion of low-dose remifentanil (0.01–0.03 μg/kg/min) were used for analgesia. Then, we were eventually able to analyze 20 patients who had no pain immediately after surgery upon arrival at PACU (NRS between 0 and 2).

All 20 patients remained in PACU for at least 12 h after surgery. In such 20 patients studied, flurbiprofen axetil 50 mg was used for analgesia if postoperative pain occurred (1 h after surgery). These 20 patients (who had no pain upon admission to PACU) were divided into two groups at 1 h after the surgery. Group A (*n* = 16) had no pain upon admission to PACU and remained pain free upon discharge from PACU (12 h after surgery); group B (*n* = 4) had no pain upon admission to PACU, but experienced increasing pain in PACU 1 h after surgery, requiring analgesic therapy.

The NRS was assessed by attending anesthesiologists who were blinded to the use of NSAIDs immediately after entering PACU, and then at 1 and 2 h after surgery.

### Measurement of heart rate variability (HRV)

Data were analyzed for HRV using Fast Fourier Transform (Check My Heart®, Daily Care Bio Medical Inc., Taiwan) [1]. HRV was recorded immediately after admission to PACU and then 2 h thereafter as a single data entry and stored on a computer. According the previous report [1], HRV parameters were estimated in the classic frequency bands of low-frequency power (LF; 0.04–0.15 Hz), high-frequency power (HF; 0.15–0.45 Hz), and LF/HF in PACU. Anesthesiologists who analyzed HRV data in the two groups were blinded as to whether NSAIDs were used or not in PACU. HRV was measured in these 20 patients immediately after entering PACU and then at 2 h after surgery.

### Statistical analysis

All data are expressed as mean ± standard deviation (SD). Mann-Whitney *U* testing was used for analyzing comparisons between groups. Statistical significance was set at *p* < 0.05. All calculations were performed on a computer running Windows 7 and Stat Mate software (GraphPad, La Jolla, CA, USA).

## Results

Table 2 shows the demographic data of the two groups. Apart from anesthetic time, there were no significant differences in demographic data between the two groups.

**Table 2:** Demographic data of the two groups

	Group A	Group B	*p* value
Age (years)	59 ± 9	63 ± 16	0.64
Height (cm)	153 ± 5	157 ± 12	0.60
Weight (kg)	57 ± 17	56 ± 9	0.96
Anesthetic time (min)	110 ± 26	77 ± 12*	0.03
Operation time (min)	69 ± 26	46 ± 10	0.10
Remifentanil dosage during surgery (mg)	0.59 ± 0.22	0.48 ± 0.20	0.37
Sevoflurane dose during surgery (mL)	32 ± 9	25 ± 3	0.17
Fluid balance (mL)	742 ± 243	575 ± 202	0.23
Blood loss (mL)	41 ± 28	23 ± 8	0.28

Demographic data of the two groups

Group A (16 patients whose postoperative pain did not increase 12 h after the surgery)

Group B (four patients whose postoperative pain increased 1 h after the surgery)

Data are expressed as means ± SD

*p < 0.05 compared with group A

Table 3 shows the HF, LF/HF, and NRS values of the two groups. There were significant differences in HF and LF/HF in group A compared with those in group B immediately after admission to PACU (HF: group A, 35.4 ± 18.1; group B, 64.2 ± 9.5*; LF/HF: group A, 2.7 ± 2.4; group B, 0.6 ± 0.2*, *p < 0.05). There was no significant difference in NRS between the two groups immediately after entering PACU.

**Table 3:** HF, LF/HF, and NRS data of the two groups immediately after admission to PACU

	Group A	Group B	*p* value
HF	35.4 ± 18.1	64.2 ± 9.5*	0.016
LF/HF	2.7 ± 2.4	0.6 ± 0.2*	0.016
NRS at immediately after admission to PACU	0.9 ± 0.8	0.3 ± 0.5	0.169

Group A (16 patients whose postoperative pain did not increase 12 h after the surgery)

Group B (four patients whose postoperative pain increased 1 h after the surgery)

Data are expressed as means ± SD

*LF* low-frequency power, *HF* high-frequency power, *NRS* numerical rating scale, *PACU* post-anesthesia care unit

*p < 0.05 compared with group A

Table 4 shows the hemodynamic data of the two groups immediately upon arrival in PACU. There were no significant differences in heart rate (HR), systolic blood pressure, diastolic blood pressure, and SpO_2_ between the two groups when assessing HRV in PACU.

**Table 4:** Hemodynamic data of the two groups at measurement of HRV immediately after admission to PACU

	Group A	Group B	*p* value
HR (beat/min)	82.8 ± 12.2	76 ± 12.7	0.42
Systolic blood pressure (mmHg)	139.9 ± 14.6	144.5 ± 16.9	0.78
Diastolic blood pressure(mmHg)	81 ± 15.9	68.5 ± 12.4	0.10
SpO2 (%)	99.4 ± 1.1	100 ± 0	0.24

Group A (16 patients whose postoperative pain did not increase 12 h after the surgery)

Group B (four patients whose postoperative pain increased 1 h after the surgery)

Data are expressed as means ± SD

*HR* heart rate

Table 5 shows the postoperative NRS of the two groups 1 h after surgery before the use of analgesic agents. NRS values at 1 h after surgery in group B were higher than that in group A (NRS: group A: 1.0 ± 0.9; group B: 4.0 ± 1.4*, *p < 0.01).

**Table 5:** Postoperative NRS of the two groups at 1 h after surgery prior the use of analgesic agents

	Group A	Group B	*p* value
NRS at 1 h prior the use of analgesia	1.0 ± 0.9	4.0 ± 1.4*	0.005

Group A (16 patients whose postoperative pain did not increase 12 h after the surgery)

Group B (four patients whose postoperative pain increased 1 h after the surgery)

Data are expressed as means ± SD

*NRS* numerical rating scale

*p < 0.05 compared with group A

Table 6 shows the HF, LF/HF, and NRS values of the two groups at 2 h after surgery. There were no significant differences in HF, LF/HF, and NRS between the two groups at 2 h after surgery. NRS values 2 h after surgery in group B decreased after the use of analgesic agents (flurbiprofen axetil 50 mg).

**Table 6:** HF, LF/HF, and NRS data of the two groups 2 h after admission to PACU

	Group A	Group B	*p* value
HF	41.3 ± 19.8	44.6 ± 14.6	0.621
LF/HF	2.6 ± 2.8	1.5 ± 1.2	0.603
NRS at 2 h admission to PACU	1 ± 0.9	0.5 ± 1	0.292

Group A (16 patients whose postoperative pain did not increase 12 h after the surgery)

Group B (4 patients whose postoperative pain increased 1 h after the surgery)

Data are expressed as means ± SD

*LF* low-frequency power, *HF* high-frequency power, *NRS* numerical rating scale, *PACU* post-anesthesia care unit

In all 16 patients in group A, no analgesic agents were required for at least 12 h since their NRSs values were kept below 3 during the 12 h after the surgery.

## Discussion

The present study revealed that lower HF and higher LF/HF values were observed immediately arrival in PACU in patients who had adequate analgesia for 12 h after the surgery concluded when compared to those who did not have adequate pain relief 1 h after surgery. This suggests that assessment of HRV may be related to the appearance of postoperative pain.

Some reports have described a possible association between changes in HRV and pain prediction. Jeanne et al. [2] reported that anesthesia reduced total power as well as HF and LF power and that HF decreased during light planes of anesthesia, whereas HRV did not change when patients received adequate analgesia. In their report, they suggested that HF might be an early indicator of inadequate analgesia. Subsequently, Papaioannou et al. [3] examined that the balance between analgesia and nociception calculated from HRV-predicted pain perception in burns patients. They reported that analgesia has been associated with a shift toward HF predominance, whereas a shift toward LF may serve as an index of increased sympathetic tone due to nociception.

A recent review article [4] showed that HRV has several advantages when compared to other measures of autonomic reactivity in studies investigating the physiological response to nociceptive stimulation. These reports led us to the hypothesis that measuring HRV could predict the manifestation of postoperative pain in surgical patients. This is the first study to examine whether measuring HRV could predict the development of postoperative pain in patients undergoing breast cancer surgery.

In this study, HRV differed between patients who had increased postoperative pain 12 h after surgery and patients whose pain remained adequately controlled. It is unclear why HRV differed between the two groups immediately after surgery, despite there being no differences in NRS in either group at that time. Possible mechanisms for consideration include that the balance of sympathetic and parasympathetic tone (which did not affect hemodynamic changes such as heart rate or blood pressure) would be altered prior to an increase in pain intensity. Broucqsault-Dédrie et al. [10] reported that HRV would be a useful tool for detecting pain in deeply sedated patients. Taneyama et al. [11] showed that HRV analysis might be a useful modality for guiding pain management. Recently, Boselli et al. [7] reported that assessment of HRV could predict the appearance of postoperative pain immediately following the end of surgery. In their study, they showed that measurement of the analgesia/nociception index derived from HRV immediately before extubation (after combined volatile and remifentanil general anesthesia) was associated with pain intensity on arrival in PACU. Our study may indicate that assessing HRV could be a useful tool for predicting the appearance of postoperative pain. The clinical implication of this study is that anesthesiologists would be able to intervene timeously to ensure adequate postoperative analgesia by using a tool that is an early predictor of postoperative surgical pain.

In our study, lower HF values were found in patients whose postoperative pain did not increase after 12 h in PACU. This is in contrast to a previous study by Papaioannou et al. [3] that showed that analgesia is associated with a shift toward HF predominance, whereas a shift toward LF may serve as an index of increased sympathetic tone secondary to nociception. Although we are unable to explain our contradictory findings, many factors may have contributed to increased postoperative pain, in addition to sympathetic tone activation [12].

As such, we need to identify the relationship between HRV changes and postoperative pain. In addition, the subjects in whom HRV was assessed were patients undergoing breast cancer surgery. These patients typically have little postoperative pain compared to patients undergoing abdominal surgery who would need intervention for severe pain before HRV could be assessed. We thus cannot rule out that differing results may be seen in other postoperative patient groups.

### Study limitations

There are several limitations to this study. Firstly, the number of patients assessed was small, so that further, higher powered studies are necessary to clarify whether HRV could be a clinical surrogate for postoperative pain in surgical patients. Secondly, we did not assess the course of changes in NRS and HRV across time; as such, the temporal relationship to direct alterations in NRS and HRV were not shown, because we focused on whether HRV could be a clinical surrogate predictive of postoperative pain in surgical patients who were pain free upon arrival in PACU. As such, we need to examine the relationship between alterations in NRS and HRV over time when postoperative pain increases. This study was also retrospective. A future, prospective study is recommended to help identify the relationship between HRV changes and postoperative pain. Ledowski et al. [13] showed possible differential relationships between HRV assessment and postoperative pain with selected anesthetic agents, such as total intravenous or volatile anesthetic agents. As such, we cannot rule out the possibility that the anesthetic agents used in this study affected our results.

## Conclusion

Lower HF and higher LF/HF values were observed immediately after arrival in PACU in patients, who had adequate analgesia for 12 h after surgery concluded, compared to patients whose pain relief was not maintained for 1 h after surgery. This suggests that differences in HRV value may be related to the appearance of postoperative pain.

## Abbreviations

HF: High-frequency power
HRV: Heart rate variability
LF: Low-frequency power
NRS: Numerical rating scale
NSAIDs: Non-steroidal anti-inflammatory drugs
PACU: Post-anesthesia care unit
PECS: Pectoral compartment block
UMIN: University Hospital Medical Information Network

## References

[1] Komatsu T, Fujiwara Y, Hashimoto A, Harato M, Ito H (2009). Analysis of heart rate variability. Masui.

[2] Jeanne M, Logier R, De Jonckheere J, Tavernier B (2009). Heart rate variability during total intravenous anesthesia: effects of nociception and analgesia. Auton Neurosci.

[3] Papaioannou V, Chouvarda I, Gaertner E, Benyamina M, Ferry A, Maurel V, Soussi S, Blet A, Chaouat M, Plaud B, Mebazaa A, Legrand M, PRONOBURN Group (2016). Heart rate variability and cardiac baroreflex inhibition-derived index predicts pain perception in burn patients. Burns.

[4] Koenig J, Jarczok MN, Ellis RJ, Hillecke TK, Thayer JF (2014). Heart rate variability and experimentally induced pain in healthy adults: a systematic review. Eur J Pain.

[5] Boselli E, Bouvet L, Bégou G, Dabouz R, Davidson J, Deloste JY, Rahali N, Zadam A, Allaouchiche B (2014). Prediction of immediate postoperative pain using the analgesia/nociception index: a prospective observational study. Br J Anaesth.

[6] Guignard B (2006). Monitoring analgesia. Best Pract Res Clin Anaesthesiol.

[7] Boselli E, Daniela-Ionescu M, Bégou G, Bouvet L, Dabouz R, Magnin C, Allaouchiche B (2013). Prospective observational study of the non-invasive assessment of immediate postoperative pain using the analgesia/nociception index (ANI). Br J Anaesth.

[8] Lindh V, Wiklund U, Håkansson S (1999). Heel lancing in term new-born infants: an evaluation of pain by frequency domain analysis of heart rate variability. Pain.

[9] Blanco R (2011). The ‘pecs block’: a novel technique for providing analgesia after breast surgery. Anaesthesia.

[10] Broucqsault-Dédrie C, De Jonckheere J, Jeanne M, Nseir S (2016). Measurement of heart rate variability to assess pain in sedated critically ill patients: a prospective observational study. PLoS One.

[11] Taneyama C, Yokota S, Goto H (2013). Patients with complex regional pain syndrome type 1: fractal dynamics of heart rate variability and baroreflex evaluations. Clin J Pain.

[12] Deumens R, Steyaert A, Forget P, Schubert M, Lavand’homme P, Hermans E, De Kock M (2013). Prevention of chronic postoperative pain: cellular, molecular, and clinical insights for mechanism-based treatment approaches. Prog Neurobiol.

[13] Ledowski T, Tiong WS, Lee C, Wong B, Fiori T, Parker N (2013). Analgesia nociception index: evaluation as a new parameter for acute postoperative pain. Br J Anaesth.

